# Medical Debt and Forgone Mental Health Care Due to Cost Among Adults

**DOI:** 10.1001/jamahealthforum.2025.0383

**Published:** 2025-04-18

**Authors:** Kyle J. Moon, Katherine E. M. Miller, Sandro Galea, Catherine K. Ettman

**Affiliations:** 1 Department of Mental Health, Johns Hopkins Bloomberg School of Public Health, Baltimore, Maryland; 2 Department of Health Policy and Management, Johns Hopkins Bloomberg School of Public Health, Baltimore, Maryland; 3 Department of Epidemiology, Boston University School of Public Health, Boston, Massachusetts; 4 Office of the Dean, Boston University School of Public Health, Boston, Massachusetts; 5 Editor, *JAMA Health Forum*

## Abstract

This cohort study evaluated the association between medical debt among US adults and forgoing mental health care due to cost in the subsequent year.

## Introduction

Affordability remains one of the biggest challenges in US health care, with a growing burden of medical debt.^1,2^ Medical debt reflects prior health care engagement but may also influence care-seeking behavior. Fewer than half of adults with any mental disorder receive care,^3^ and medical debt may exacerbate this treatment gap.^4^ This study evaluated the association between medical debt and forgone mental health care due to cost in the subsequent year.

## Methods

A nationally representative cohort of US adults was surveyed in 2023 through 2024 through the COVID-19 Life Stressors Impact on Mental Health and Well-Being (CLIMB) study.^5^ Of the 7802 adults invited to participate, 2479 (31.8%) responded in 2023, of whom 2020 (81.5%) participated in 2024. For the present cohort study, we limited the sample to 1821 respondents with no missing data for the primary exposure (medical debt) and outcome (forgone mental health care due to cost) and used multiple imputation by chained equations for missing covariates (n = 100), generating 20 imputed datasets. This study was deemed exempt from review by the Johns Hopkins institutional review board with a waiver of informed consent because it was not human participant research. We performed analyses using R, version 4.3.1 (R Project for Statistical Computing) and followed the STROBE guideline.

We fit survey-weighted logistic regression models estimating forgone mental health care as a function of medical debt, first modeling debt as a dichotomous exposure (none vs any), then as a categorical measure, and adjusted for sociodemographic characteristics. To account for the temporality of the exposure and outcome, we used medical debt reported in 2023 and forgone mental health care in 2024. We reported results with unweighted frequencies, weighted percentages, and average marginal effects (eMethods in Supplement 1).

## Results

Among 1821 adults (mean [SD] age, 51.6 [16.7] years; 805 female [50.5% weighted]), 276 (15.3% weighted) reported medical debt in 2023. Forgone mental health care was significantly higher among adults with past-year medical debt (33.8% vs 6.3% weighted). Any medical debt was associated with an increase of 17.3 (95% CI, 11.8-22.8) percentage points in the probability of forgone mental health care due to cost, with an increase in the probability of unmet mental health care needs with increasing medical debt (Table).

**Table.** Probability of Forgone Mental Health Care Due to Cost Among US Adults in 2024

Medical debt	Adults, unweighted No.	Prevalence of forgoing mental health care due to cost, weighted % (95% CI)	Average marginal effect, percentage points (95% CI)^a^			
			Crude model	*P* value^b^	Adjusted model^a^	*P* value^b^
No	1545	6.3 (5.2-8.0)	0 [Reference]	NA	0 [Reference]	NA
Yes	276	33.8 (27.8-40.0)	27.5 (21.0-33.9)	<.001	17.3 (11.8-22.8)	<.001
Amount, $						
<1000	121	28.9 (18.6-42.0)	22.6 (11.1-34.1)	<.001	11.7 (4.4-19.0)	.002
1000-4999	107	33.1 (24.9-43.0)	26.8 (18.4-35.3)	<.001	17.6 (10.4-24.7)	<.001
≥5000	48	46.3 (29.7-64.0)	40.0 (24.4-55.6)	<.001	28.1 (15.6-40.6)	<.001

Abbreviation: NA, not applicable.

^a^ Model was adjusted for participant sex, age, race, ethnicity, educational attainment, employment, annual household income, household savings, household size, geographic division of residence, and metropolitan statistical area of residence.

^b^ The threshold for statistical significance was *P* < .05.

## Discussion

More than 1 in 7 adults reported carrying medical debt in 2023, of whom 1 in 3 forwent mental health care in the subsequent year. Medical debt may exacerbate the treatment gap^4^ by potentially (1) raising the threshold for seeking care, (2) eroding patient trust in the health system, or (3) being denied care due to outstanding debts.

This study has several limitations. We cannot draw causal inferences, and our primary exposure was self-reported, making it subject to recall bias. While prevalence estimates of medical debt align with prior studies,^4^ respondents may underestimate debt amount due to surprise billing, lapses in insurance, or interest fees.^2^ However, perceived medical debt may affect care-seeking behavior to a greater extent than actual debt. Additionally, despite using survey weights to account for sampling probabilities and nonresponse, there is still potential for nonresponse bias in our prevalence estimates. We found some evidence that medical debt amount may modify the association with forgone care, but CIs were overlapping, likely due to limited statistical power. Further research should consider longer time horizons and evaluate how both insurance and debt amount may modify the association between medical debt and forgone mental health care.

While there are a constellation of factors leading to unmet needs for mental health care,^3^ medical debt is an iatrogenic problem that leaves patients grappling with the decision to pay large out-of-pocket costs, accumulate medical debt, or forgo needed care.^6^ Several policy efforts to address medical debt are underway, and there remains an urgent need to understand how these interventions protect against medical debt and if such protections can aid in addressing unmet needs for mental health care.

## Notes

Supplement 1. eMethods

Supplement 2. Data Sharing Statement
