# Supplementary material for: Medical Debt and Forgone Mental Health Care Due to Cost Among Adults
Source: JAMA Health Forum. 2025 Apr 18;6(4):e250383. doi: 10.1001/jamahealthforum.2025.0383 (PMC12008752; doi:10.1001/jamahealthforum.2025.0383)
Supplement: Supplement 1. — eMethods [file jamahealthforum-e250383-s001.pdf]

Supplemental Online Content

Moon KJ, Miller KEM, Galea S, Ettman CK. Medical debt and foregone mental health care due to cost among adults. *JAMA Health Forum*. 2025;6(4):e250383. doi:10.1001/jamahealthforum.2025.0383

eMethods
eReferences

This supplemental material has been provided by the authors to give readers additional information about their work.

© 2025 Moon KJ et al. *JAMA Health Forum*.

eMethods

This cohort study analyzes data from the fourth and fifth waves of the COVID-19 Life Stressors Impact on Mental Health and Well-Being (CLIMB) study, a nationally representative, longitudinal panel of U.S. adults. CLIMB participants are recruited from the AmeriSpeak panel conducted by NORC at the University of Chicago using non-zero probability-based methods from an address-based sampling frame that encompasses 97% of U.S. households. The fourth and fifth waves of the CLIMB survey were fielded in March–April 2023 and March–April 2024, respectively. All participants were ≥18 years of age and provided informed consent before initiating the survey. Survey responses were weighted to align the study sample with the U.S. adult population based on the U.S. Current Population Survey for age, gender, Census Division, Race/Ethnicity, Education, Age x Gender, Age x Race/Ethnicity, Race/Ethnicity x Race based on the past year's March Current Population Survey (CPS). Demographic representativeness of the panel has been assessed against the CPS, U.S. Census Bureau's American Community Survey, and National Center for Health Statistics National Health Interview Survey.[1]

Our primary exposure of interest, medical debt, was ascertained with the following question, "In the past 12 months, did you have problems paying or an inability to pay any medical bills, such as bills for doctors, dentists, medication, or home care? Please include any bills you have had problems paying over the past 12 months, even if the initial bill was incurred more than 12 months ago," consistent with other work.[2] Those that endorsed past-year medical debt were asked to estimate the total amount that they personally owed for medical bills, not including any amount paid by insurance. Our primary outcome of interest was foregone mental health care due to cost, which was defined as an endorsement of the survey item, "Was there a time in the past 12 months when you wanted to see a health provider about your mental health but could not because you could not afford it?"

Sociodemographic characteristics, which included sex (male or female), age, race (Asian, Black or African American, White, or other), ethnicity (Hispanic or Latino, non-Hispanic

© 2025 Moon KJ et al. *JAMA Health Forum*.

or Latino), educational attainment, employment status, health insurance, annual household income, household savings, and household size, were self-reported by the participant. Geographic characteristics of the respondent, including U.S. Census division (New England, Middle Atlantic, East North Central, West North Central, South Atlantic, East South Central, West South Central, Mountain, and Pacific) and metropolitan statistical area (MSA) designation, were recorded by NORC at the University of Chicago.

By accounting for survey weights and the complex survey design of CLIMB, we computed weighted percentages with corresponding 95% confidence intervals (CIs) using Taylor series linearization. We fit a series of survey-weighted logistic regression models that predicted the probability of foregone mental health care needs due to cost as a function of medical debt. We first treated medical debt as a dichotomous exposure (none vs. any), fitting one crude and one adjusted model. We then fit crude and adjusted models using a categorical measure of medical debt amount: none (reference), < $1,000 in medical debt, $1,000–4,999 in medical debt, and ≥ $5,000 in medical debt. While marginal effects are used in both observational and experimental studies, our use of the term "effect" does not imply causality. All adjusted models included the following covariates: sex, age, race, ethnicity, educational attainment, employment status, annual household income, household savings, household size, geographic region, and MSA designation. We did not include health insurance due to endogeneity concerns, but we fit separate models that included health insurance as a covariate as a robustness check. We find no evidence of multicollinearity (variance inflation factors ≤ 2.69), heteroskedasticity (White test-statistic = 2, P = 0.30), or autocorrelation (Durbin-Watson test-statistic = 2, P = 0.10) in any of our models.

Among the AmeriSpeak panelists invited to take the CLIMB survey in 2023, 31.8% completed the survey; among this group, 81.5% were retained the subsequent year. This response rate is comparable to rates reported in other national surveys,[3,4] as previously described.[5] Of the 1,821 adults in our analytic sample, 100 (5%) had missing data for one or

© 2025 Moon KJ et al. *JAMA Health Forum*.

more of the following covariates: health insurance (n = 10), employment status (n = 8), annual household income (n = 48), household savings (n= 73), and/or household size (n = 22). We used multiple imputation by chained equations (MICE) to impute missing values, generating 20 imputed datasets with the mice package in R Statistical Software.[6] Five iterations were performed. All covariates with missing values were nominal (i.e., non-ordinal) categorical variables, so we used multinomial logit models. We applied Rubin's rules to pool estimates and compute standard errors that account for between-imputation and within-imputation variance. We assessed the imputation procedure by comparing the marginal effect and confidence interval estimates obtained from (a) multiple imputation and (b) complete case analysis, which yielded comparable results.

eReferences

1. *AmeriSpeak® Panel Demographics Report*.; 2024. Accessed December 21, 2024. https://amerispeak.norc.org/content/dam/amerispeak/research/pdf/AmeriSpeak%20Panel%20Demographic%20Report.pdf
2. Moon KJ, Linton SL, Mojtabai R. Medical Debt and the Mental Health Treatment Gap Among US Adults. *JAMA Psychiatry*. Published online July 17, 2024. doi:10.1001/jamapsychiatry.2024.1861
3. Williams CP, Senft Everson N, Shelburne N, Norton WE. Demographic and Health Behavior Factors Associated With Clinical Trial Invitation and Participation in the United States. *JAMA Netw Open*. 2021;4(9):e2127792. doi:10.1001/jamanetworkopen.2021.27792
4. Duffy EL, Frasco MA, Trish E. Disparate Patient Advocacy When Facing Unaffordable and Problematic Medical Bills. *JAMA Health Forum*. 2024;5(8):e242744. doi:10.1001/jamahealthforum.2024.2744
5. Holman EA, Thompson RR, Garfin DR, Silver RC. The unfolding COVID-19 pandemic: A probability-based, nationally representative study of mental health in the United States. *Sci Adv*. 2020;6(42). doi:10.1126/sciadv.abd5390
6. Buuren S van, Groothuis-Oudshoorn K. mice: Multivariate Imputation by Chained Equations in R. *J Stat Softw*. 2011;45(3). doi:10.18637/jss.v045.i03

© 2025 Moon KJ et al. *JAMA Health Forum*.
