# Supplementary material for: Medical Debt and Forgone Mental Health Care Due to Cost Among Adults
Source: JAMA Health Forum. 2025 Apr 18;6(4):e250383. doi: 10.1001/jamahealthforum.2025.0383 (PMC12008752; doi:10.1001/jamahealthforum.2025.0383)
Supplement: Supplement 2. — Data Sharing Statement [file jamahealthforum-e250383-s002.pdf]

Data Sharing Statement

Moon. Medical Debt and Foregone Mental Health Care Due to Cost Among Adults. *JAMA Health Forum*. Published April 18, 2025. doi:10.1001/jamahealthforum.2025.0383

Data
Data available: Yes
Data types: Deidentified participant data, Data dictionary
How to access data: Data are available upon reasonable request for research purposes sent to CKE: cettman1@jhu.edu.
When available: With publication

Supporting Documents
Document types: None

Additional Information
Who can access the data: Researchers whose proposed use of the data has been approved.
Types of analyses: For specific research analyses approved by study team.
Mechanisms of data availability: After approval of a proposal and signed data access agreement.
